# *Tropheryma whipplei* detection by metagenomic next-generation sequencing in bronchoalveolar lavage fluid: A cross-sectional study

**DOI:** 10.3389/fcimb.2022.961297

**Published:** 2022-08-17

**Authors:** Minmin Lin, Kongqiu Wang, Lidi Qiu, Yingjian Liang, Changli Tu, Meizhu Chen, Zhenguo Wang, Jian Wu, Yiying Huang, Cuiyan Tan, Qijiu Chen, Xiaobin Zheng, Jing Liu

**Affiliations:** ^1^ Department of Pulmonary and Critical Care Medicine (PCCM), the Fifth Affiliated Hospital of Sun Yat-sen University, Zhuhai, China; ^2^ Guangdong Provincial Key Laboratory of Biomedical Imaging and Guangdong Provincial Engineering Research Center of Molecular Imaging, The Fifth Affiliated Hospital of Sun Yat-sen University, Zhuhai, China; ^3^ Department of Infectious Disease Intensive Care Unit, The Fifth Affiliated Hospital of Sun Yat-sen University, Zhuhai, China

**Keywords:** *Tropheryma whipplei*, metagenomic next-generation sequencing, bronchoalveolar lavage fluid, Mycobacterium tuberculosis complex, pulmonary nodules

## Abstract

*Tropheryma whipplei* is the bacterium associated with Whipple’s disease (WD), a chronic systemic infectious disease primarily involving the gastrointestinal tract. *T. whipplei* can also be detected in different body site of healthy individuals, including saliva and feces. Traditionally, *Tropheryma whipplei* has a higher prevalence in bronchoalveolar lavage fluid (BALF) of immunocompromised individuals. Few studies have explored the significance of the detection of *T. whipplei* in BALF. Herein, we retrospectively reviewed 1725 BALF samples which detected for metagenomic next-generation sequencing (mNGS) from March 2019 to April 2022 in Zhuhai, China. Seventy BALs (70/1725, 4.0%) from 70 patients were positive for *T. whipplei.* Forty-four patients were male with an average age of 50 years. The main symptoms included cough (23/70), expectoration (13/70), weight loss (9/70), and/or dyspnea (8/70), but gastrointestinal symptoms were rare. Chronic liver diseases were the most common comorbidity (n=15, 21.4%), followed by diabetes mellitus (n=13, 18.6%). Only nine patients (12.9%) were immunocompromised. Twenty-four patients (34.3%) were finally diagnosed with reactivation tuberculosis and 15 patients (21.4%) were diagnosed with lung tumors, including 13 primary lung adenocarcinoma and two lung metastases. Fifteen patients (21.4%) had pneumonia. Among the 20 samples, *T. whipplei* was the sole agent, and Mycobacterium tuberculosis complex was the most common detected other pathogens. Among the non-tuberculosis patients, 31 (31/46, 67.4%) had ground glass nodules or solid nodules on chest CT. Our study indicates that *T. whipplei* should be considered as a potential contributing factor in some lung diseases. For non-immunocompromised patients, the detection of *T. whipplei* also needs attention. The mNGS technology improves the detection and attention of rare pathogens. In the future, the infection, colonization, and prognosis of *T. whipplei* in lung still need to be studied.

## Introduction

*Tropheryma whipplei* is the bacterium associated with Whipple’s disease (WD), described by George H.Whipple in 1907 (GH, 1907). Whipple’s disease is a rare, chronic, and systemic illness, which mainly involves the gastrointestinal tract. It can also lead to the involvement of joints, nervous system, and cardiovascular system. The most common symptoms of patients with classic Whipple’s disease are arthralgia, diarrhea, weight loss, lymphadenopathy, abdominal pain, and fever (Dolmans et al., 2017). Only a few studies on the prevalence and incidence rate of Whipple’s disease were performed. According to a research from the United States, the overall prevalence of Whipple’s disease in the US was 9.8 cases per 1 million people (Elchert et al., 2019). Of concern, the classic Whipple’s disease is typically found in Caucasian populations, but carriage is common in the native Asian and Africa populations (Keita et al., 2015).

*T. whipplei* is a rod-shaped, Gram-positive bacterium belonging to *Actinomycetes*. With the development of molecular biology technology, the bacterial 16S ribosomal DNA was first identified in small-bowel biopsy specimens of patients with classic Whipple’s disease by nucleotide sequencing and PCR amplification in 1991 (Relman et al., 1992). Subsequently, the *T. whipplei* DNA has been detected in various samples by using PCR, including stool (Schoniger-Hekele et al., 2007), saliva, urine, blood, cerebrospinal fluid, and bronchoalveolar lavage fluid (BALF) (Fenollar et al., 2012). In some studies, *T. whipplei* has been shown to be an etiological pathogen in pneumonia (Bousbia et al., 2010). Moreover, *T. whipplei* was significantly more common in BALF in HIV-positive individuals, which was considered to be a potential contributing factor with lung complications (Lozupone et al., 2013). This further indicates that *T. whipplei* may be involved in the occurrence of some lung diseases.

With the application of metagenomic next-generation sequencing (mNGS) technology with high sensitivity in our country, the detection of *T. whipplei* has increased. However, at present, the epidemiology of *T. whipplei* in BALF in our country is still lacking. Therefore, the BALF samples which detected for mNGS in our hospital were reviewed, and the characteristics of patients with positive *T. whipplei* were analyzed.

## Material and methods

### Study patients and cases definition

We retrospectively reviewed 1725 BALF samples which detected for metagenomic next-generation sequencing from March 2019 to April 2022 at the Fifth Affiliated Hospital of Sun Yat-sen University in Zhuhai, China. The collection process of BALF was in line with clinical operation standard and followed the principle of sterility. The involved sub-segments were washed with 20 to 50ml normal saline. 3-5ml BALF samples were placed in sterile sputum containers, stored in -20°C, and then sent to BGI (Shenzhen, China) for detection.

The baseline data on patients with positive *Tropheryma whipplei* was collected, including demographic information, laboratory data, clinical symptoms, imaging examination results, diagnosis, pathological results, and treatment history. Immunocompromised status was defined as any of the following: (1) solid organ transplantation; (2) long-term therapy with corticosteroid (≥ 20mg prednisone or equivalent daily for ≥ 14d or a cumulative dose > 600mg of prednisone) or other immunosuppressive drugs; (3) agranulocytosis after cancer chemotherapy; (4) hematological malignancy; (5) inherited or acquired severe immunodeficiency or human immunodeficiency virus infection.

### DNA extraction and mNGS sequencing

1.5mL microcentrifuge tube with 0.6mL sample and 250μL 0.5mm glass bead were attached to a horizontal platform on a vortex mixer and agitated vigorously at 2800-3200 rpm for 30 min. Then 7.2μL lysozyme was added for wall-breaking reaction. 0.3mL sample was separated into a new 1.5mL microcentrifuge tube and DNA was extracted using the TIANamp Micro DNA Kit (DP316, TIANGEN BIOTECH) according to the manufacturer’s recommendation. DNA libraries were constructed through DNA-fragmentation, end-repair, adapter-ligation and PCR amplification. Agilent 2100 was used for quality control of the DNA libraries. Quality qualified libraries were pooled, DNA Nanoball (DNB) was made and sequenced by MGISEQ-2000 platform.

### Bioinformatic analysis

High-quality sequencing data were generated by removing low-quality reads, followed by computational substraction of human host sequences mapped to the human reference genome (hg19) using Burrows-Wheeler Alignment (Li and Durbin, 2009). The remaining data by removal of low-complexity reads were classified by simultaneously aligning to Pathogens metagenomics Database (PMDB), consisting of bacteria, fungi, viruses and parasites. The classification reference databases were downloaded from NCBI (ftp://ftp.ncbi.nlm.nih.gov/genomes/). RefSeq contains 4,945 whole genome sequence of viral taxa, 6,350 bacteral genomes or scaffolds, 1064 fungi related to human infection, and 234 parasites associated with human diseases. *T. whipplei* was considered positive when at least 3 reads were mapped to the species level.

### Statistical analysis

Categorical variables were expressed as percentages. Continuous variables subject to normal distribution were expressed by mean and SD, otherwise, median and IQR. Comparative analysis was conducted by t test, Pearson’s test, Fisher’s exact test, or Mann-Whitney U test where appropriate. Data analysis was performed with SPSS 20.0 and GraphPad Prism. *P* < 0.05 was considered significant.

## Results

### Clinical presentation

Seventy BALs (70/1725, 4.0%) from 70 patients were positive for *T. whipplei.* Forty-four patients were male (62.9%). The age of the 70 patients ranged from 25 to 78 years (mean age: 50 years). All patients were hospitalized. Twenty-eight (40%) patients were hospitalized in infectious diseases department, 24 (34.3%) in thoracic surgery, 15 (21.4%) in respiratory department, two in an intensive-care unit and one in endocrine department at the sampling time of BALF. The distribution of sampling time was dispersed. The main clinical symptoms of patients included cough (23/70), expectoration (13/70), weight loss (9/70), and/or dyspnea (8/70) (**Table 1**). Only nine patients complained about gastrointestinal symptoms such as abdominal pain, diarrhea, vomiting, or poor appetite. One patient had joint pain, and one had neurological symptoms. Nearly half of the patients (n=32) had no clinical symptoms.

**Table 1:** The characteristics of patients with *T. whipplei* positive in BALF.

Parameters	*T. whipplei* positive (n=70)
Age (mean, SD)	50 (11.18)
Male	44 (62.9%)
Sampling year^a^	
2019	9 (12.9%)
2020	24 (34.3%)
2021	25 (35.7%)
2022	12 (17.1%)
BMI (mean, SD)	23.17 (3.67)
Smoking history	21 (30%)
Immunocompromised	9 (12.9%)
**Clinical symptoms**	
Cough	23 (32.9%)
Expectoration	13 (18.6%)
Weight loss	9 (12.9%)
Dypnea	8 (11.4%)
Fever	7 (10%)
Chest pain	4 (5.7%)
Abdominal pain	4 (5.7%)
Diarrhea	3 (4.3%)
Vomiting	3 (4.3%)
Hemoptysis	2 (2.9%)
Arthralgia	1 (1.4%)
Neurological	1 (1.4%)
**Blood test result**	
White blood cell count (×10^9/L) (mean, SD)	6.2 (2.05)
Lymphocyte count (×10^9/L) (mean, SD)	1.58 (0.58)
Haemoglobin (g/L) (mean, SD)	132 (21.4)
Platelet count (×10^12/L) (mean, SD)	219 (72.7)
CRP (mg/L) (median, IQR)	22.54 (1-11.12)
Erythrocyte sedimentation rate (mm/H) (median, IQR)	29.8 (5-47)

a The collection of sample began in March 2019, and ended in April 2022.

### Comorbidity, immune status, and inflammatory response

**Figure 1:**
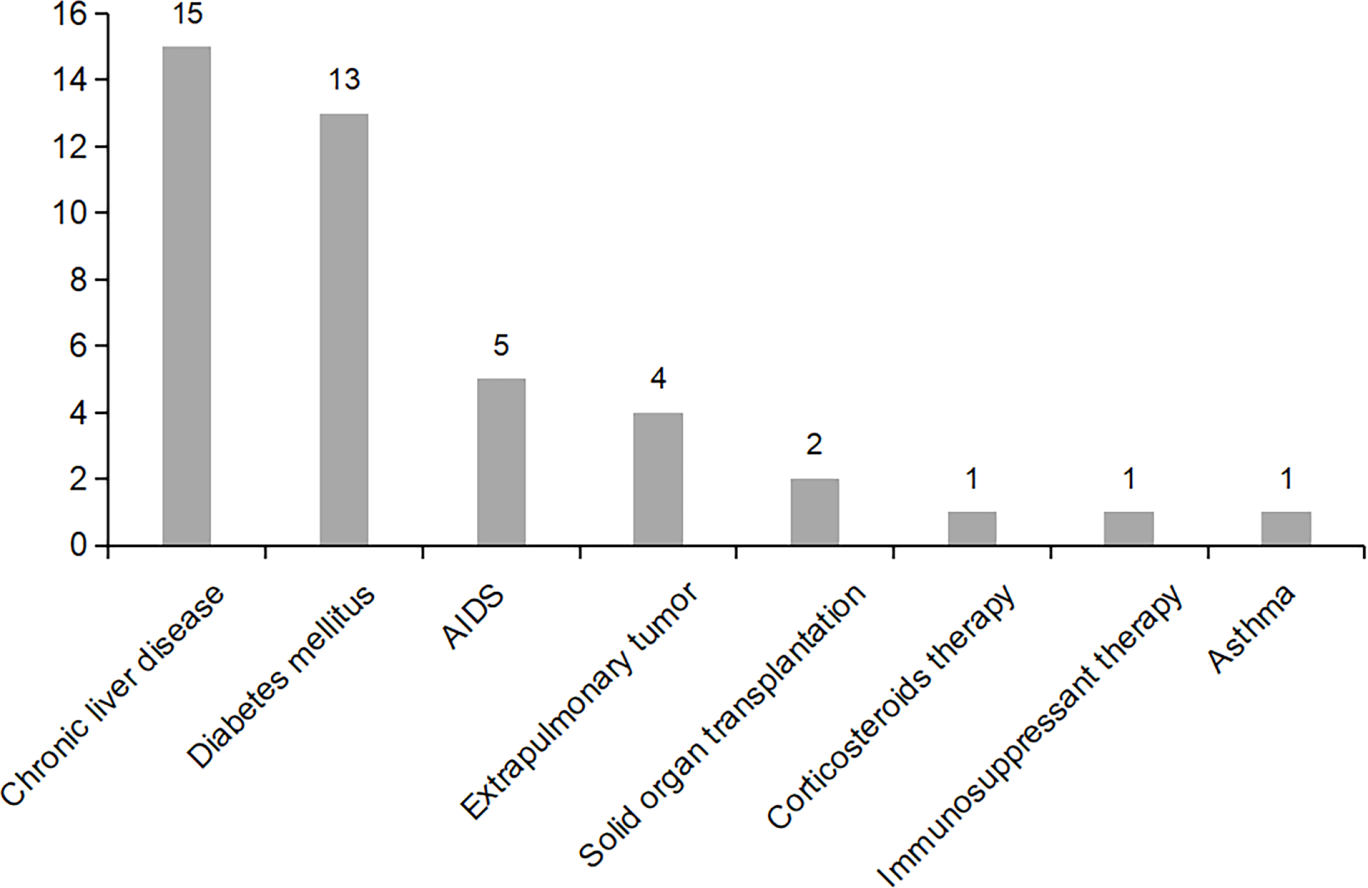
Distribution of major comorbidities in patients with positive *T. whipplei* in BALF.

Chronic liver diseases were the most common comorbidity (n=15, 21.4%), including hepatitis B, cirrhosis and fatty liver, followed by diabetes mellitus (n=13, 18.6%). Non-immunocompromised patients accounted for the majority. Immunodeficiency in nine patients (12.9%) was due respectively to drug including cyclosporin (n=1) or corticosteroids (n=1), and to medical conditions including HIV infection (n=5) and solid organ transplantation (n=2) (**Figure 1**). Four patients had extrapulmonary tumors. Almost all patients were tested for relevant inflammatory indicators after admission and before treatment. The overall inflammatory response was not strong. The average white blood cell count and haemoglobin of all 70 patients was 6.2×10^9/L (IQR 5.06-7.09×10^9/L) and 132 g/L (IQR 120-149 g/L) respectively. The median C-reactive protein was 3.5 mg/L (IQR 1-11.12 mg/L) (**Table 1**). The overall BMI index was normal (mean: 23.17, SD: 3.67), and patients with tuberculosis had a lower BMI (21.35 vs 24.13, *p*=0.002).

### Histological presentation

Twenty-one patients underwent surgery, three underwent transbronchial lung biopsy and three underwent percutaneous lung biopsy. Therefore, a total of 27 patients had lung histopathological results. The histopathological results of 15 patients were tumors, including primary lung adenocarcinoma (n=13), metastatic intestinal adenocarcinoma (n=1) and metastatic esophageal squamous cell carcinoma (n=1). Two patients showed multinucleated giant cells in histopathology, which were considered to be tuberculosis; One patient’s histopathology was positive for PAS and silver hexamine staining, which was considered as cryptococcal infection. The remaining histopathological results included nonspecific inflammatory cell infiltration (n=5), hamartoma (n=2), suspected lung cancer (n=1), and suspected multifocal micronodular alveolar epithelial hyperplasia (MMPH) (n=1).

Whipple’s disease often involves the gastrointestinal tract. Therefore, we collected the gastroscopic results. In the lamina propria of the duodenal (as well as the gastric antral region, jejunum, or ileum), foam macrophages containing large amounts of diastase-resistant PAS-positive particles was the typical histological detection. Six patients underwent gastroscopy within three months, which showed various degrees of gastritis. Other gastroscopic findings included gastric antrum ulcer (n=1), duodenal erosion and superficial ulcer (n=1), gastric mucosal intraepithelial neoplasia (n=1), gastric polyp (n=1) and esophageal cancer (n=1). Pathological biopsy of duodenal lesions showed lymphocyte and plasma cell infiltration, but PAS staining was not performed.

### mNGS and microbiological association

**Figure 2:**
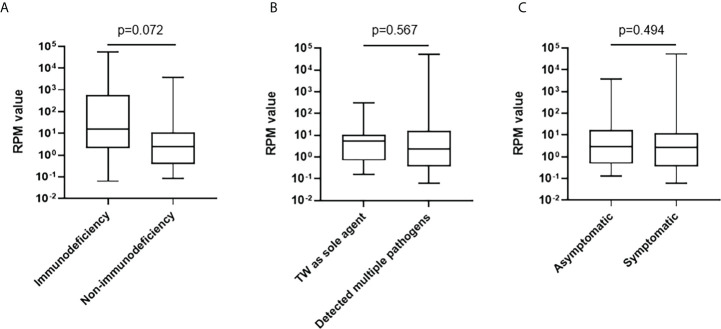
Comparisons of the RPM value of *T. whipplei* according to immune deficiency status **(A)**, mixed detection **(B)**, and clinical symptoms **(C)**.

Among 20 BALF samples, *T. whipplei* was the only pathogen detected. The most common detected bacterial pathogen with *T. whipplei* was Mycobacterium tuberculosis complex, followed by *Streptococcus pneumoniae*, *Haemophilus influenzae*, *Staphylococcus aureus*, *Haemophilus parainfluenzae*, and *Actinomyces*. The most common detected fungi was *Candida albicans*, followed by *Pneumocystis jirovecii*. Human gamma-herpes virus 4 (Epstein-Barr virus, EBV) was the most detected virus (**Table 2**). The mapped reads number of *T. whipplei* was normalized to reads per million (RPM). The RPM value of *T. whipplei* in immunodeficiency patients outnumbered those in non-immunodeficiency patients, but it did not reach statistical difference. The RPM value of *T. whipplei* sequences was not related to whether it was the sole agent and whether the patients had symptoms (**Figure 2** **)**. Immunodeficient patients had higher detection rate of fungi and virus, but only virus reached statistical difference (66.7% vs 18.0%, *p*=0.005) (**Supplementary Figure S1**). The detection rate of Mycobacterium tuberculosis complex in patients with diabetes was significantly higher (38.5% vs 8.8%, *p*=0.015).

**Table 2:** Pathogen detected by mNGS.

n=70	
*T. whipplei* as sole agent	20 (28.6%)
Mycobacterium tuberculosis complex	10 (14.3%)
*Streptococcus pneumoniae*	7 (10.0%)
*Haemophilus influenzae*	7 (10.0%)
*Staphylococcus aureus*	7 (10.0%)
*Haemophilus parainfluenzae*	5 (7.1%)
*Actinomyces*	5 (7.1%)
*Klebsiella pneumoniae*	4 (5.7%)
*Moraxella catarrhalis*	4 (5.7%)
Non-tuberculosis mycobacteria	3 (4.3%)
*Corynebacterium striatum*	1 (1.4%)
*Enterococcus faecalis*	1 (1.4%)
*Legionella*	1 (1.4%)
*Klebsiella variicola*	1 (1.4%)
*Candida albicans*	5 (7.1%)
*Pneumocystis jirovecii*	4 (5.7%)
*Candida parapsilosis*	1 (1.4%)
*Aspergillus fumigatus*	1 (1.4%)
Human gamma-herpes virus 4 (EBV)	9 (12.9%)
Human beta-herpes virus 5 (CMV)	2 (2.9%)

### Chest CT findings

**Figure 3:**
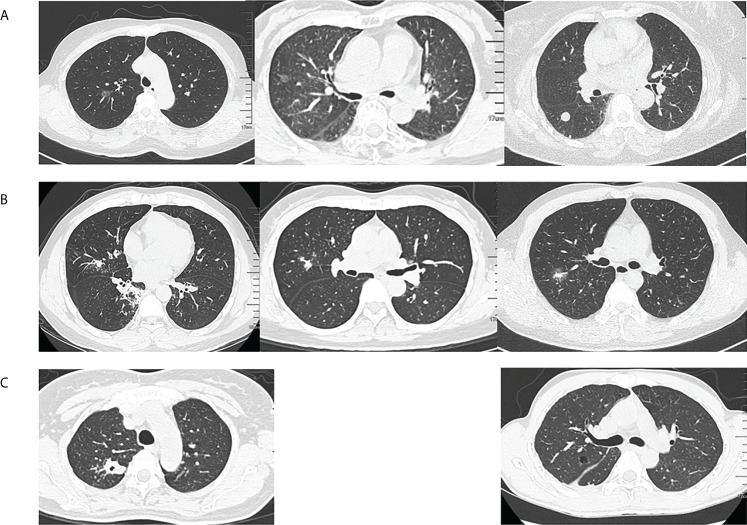
Classification of chest computed tomography (CT) in non-tuberculosis patients. **(A)** Nodular type: ground glass nodules or solid nodules, **(B)** Pneumonia type: focal or patchy mixed density shadow, and **(C)** Mixed type: other manifestations such as cavity, cystic or pleural effusion.

According to the classification of diseases, we simply described the chest CT findings. Among the tuberculosis (TB) patients, the most chest CT findings were common reactivation TB changes, including focal or patchy heterogeneous consolidation, poorly defined nodules or linear opacities, cavity (n=6), tuberculoma (n=1), and pleural effusion (n=1). One patient’s chest CT showed miliary tuberculosis. Among the non-TB patients, 31 (31/46, 67.4%) had ground glass nodules or solid nodules on chest CT. The remaining CT findings included patchy, mass, pleural effusion (n=6), cystic (n=2), cavity (n=2), and interstitial fibrosis (n=2). Classification of chest CT in non-tuberculosis patients, especially considering the *T. whipplei* as the main pathogen, it could be roughly divided into nodular type, pneumonia type and mixed type, as shown in **Figure 3**. In general, the chest imaging manifestations were various.

### Diagnosis, treatment, and prognosis

**Figure 4:**
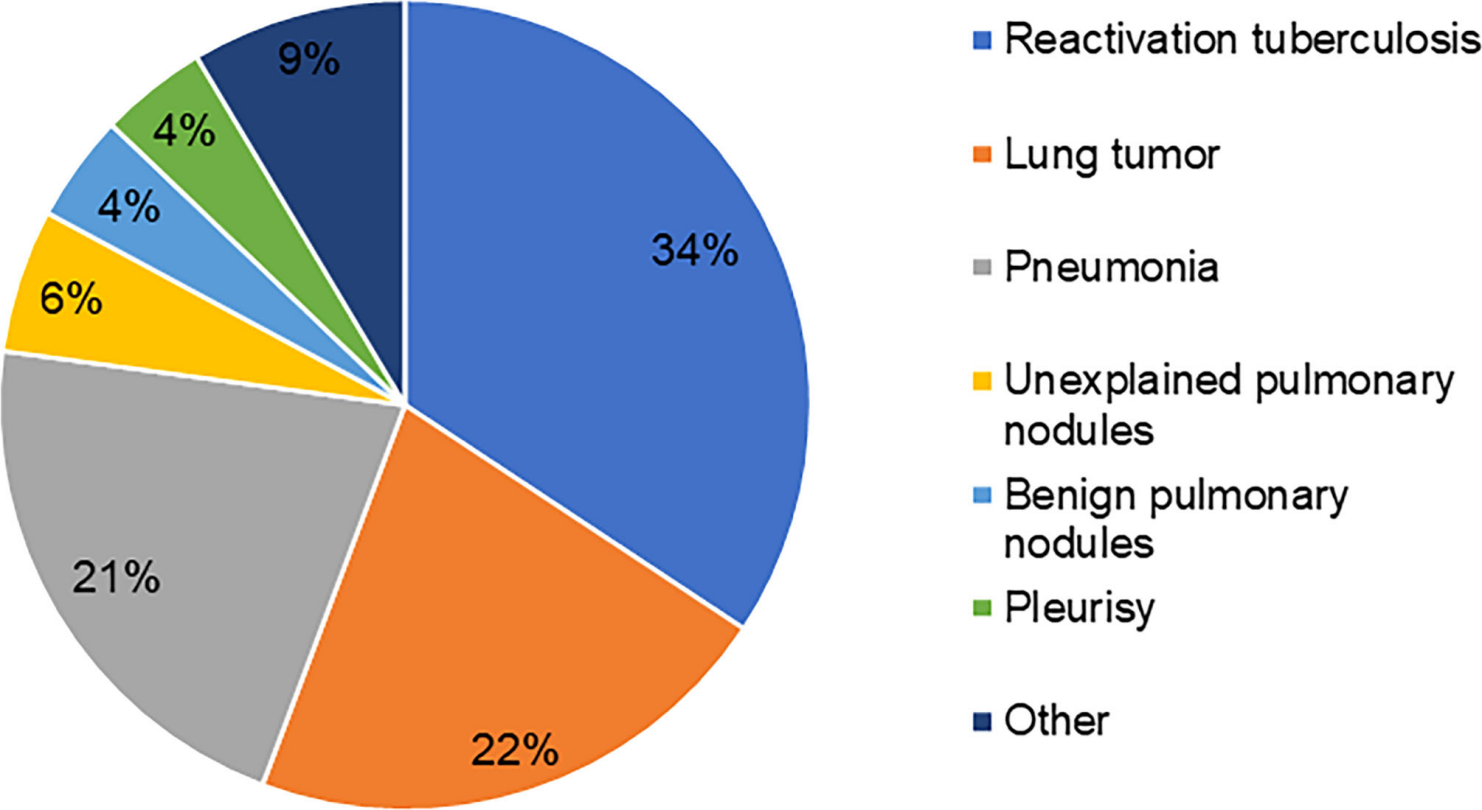
Diagnostic distribution of *T. whipplei* positive patients in BALF.

Twenty-four patients (34.3%) were finally diagnosed with reactivation tuberculosis and received anti-tuberculosis treatment. Sixteen of them improved after treatment. Fifteen patients (21.4%) were diagnosed with pneumonia, and all received antimicrobial treatment, such as β-lactams/β-lactamase inhibitor, Trimethoprim-sulfamethoxazole, doxycycline or quinolones. Six patients improved radiographically after treatment. More immunocompromised patients were diagnosed with pneumonia (*p*=0.001). According to histopathology, fifteen patients (21.4%) were diagnosed with lung tumors, including 13 primary lung adenocarcinoma and two lung metastases. Fourteen of them received surgery and one received chemotherapy. The remaining diagnoses include unexplained pulmonary nodules (n=4), benign pulmonary nodules (n=3), pleurisy (n=3), cryptococcal infection (n=1), and suspected lung cancer (n=1) (**Figure 4**). None of the patients died in hospital.

## Discussion

Although WD was described as early as 1907, *T. whipplei* was only successfully cultured in macrophages until 1997 (Schoedon et al., 1997). Due to the lack of specificity of the clinical manifestations, and the lack of understanding of WD, the disease was often misdiagnosed or undiagnosed in our country. With the development of molecular methods and application of mNGS technology, the number of positive cases detected in BALF increased. Meanwhile, there was an increasing number of cases reported about pulmonary infection caused by *T. whipplei* or pulmonary involvement of WD in recent years. However, the significance of *T. whipplei* detection in BALF still needs to be comprehensively considered. Our study found that the majority of *T. whipplei* positive patients in BALF were non-immunodeficient, with a large proportion of patients diagnosed with tuberculosis, lung tumors, and pneumonia. Pulmonary nodules were the main manifestation of chest CT. To our knowledge, our study has the largest sample size in the epidemiological study of *T. whipplei* detected in BALF in China.

In this study, mNGS was used to detect *T. whipplei*. In the process of sequencing and bioinformatics analysis, the technology has strictly set up positive and negative controls and carried out quality control. The distribution of departments during the patients sampling was relatively scattered, and the collection time was evenly distributed. Therefore, the possibility of nosocomial infection or outbreak could be ruled out, and the contamination in the collection process or laboratory process could also be basically ruled out. Accordingly, we consider the detected results to be credible.

The prevalence of *T. whipplei* in our study was slightly lower than that reported by Lagier et al. (6.1%, 88/1438) (Lagier et al., 2016), which was close to the 3% (6/210) reported by Boubia et al. (2010). Respiratory symptoms, weight loss and fever were the main clinical manifestations of patients, while gastrointestinal symptoms were rare, which was similar with the previous case reports of pneumonia caused by *T. whipplei* (Urbanski et al., 2012; Guo et al., 2021). However, nearly half of the patients in our study were asymptomatic, and the overall inflammatory response was not strong. This suggests that some patients may be carriers of *T. whipplei* in the lungs. Our results indicated that non-immunodeficient status was predominant in *T. whipplei* positive patients in BALF, which was inconsistent with the previous view that *T. whipplei* was more prevalent among HIV-positive individuals (Lozupone et al., 2013; Garcia-Alvarez et al., 2016). The case-control study of Lagier et al. found that the prevalence of *T. whipplei* may not be related to immune status (Lagier et al., 2016). In addition, our results suggest that patients with diabetes and chronic liver disease may be susceptible to *T. whipplei* in the lungs, which has not been concerned in previous studies. The significance and causes of *T. whipplei* detection in non-immunocompromised patients still need to be further explored.

Our study also confirm that *T. whipplei* is an etiologic pathogen in acute respiratory infections (Lagier et al., 2017). *T. whipplei* could be co-infected with *Pneumocystis jirovecii* and *Candida albicans*, as reported in previous cases (Li et al., 2021; Yan et al., 2021), or leaded to pulmonary infection alone (Guo et al., 2021). Moreover, we also found that immunocompromised patients were more likely to be diagnosed with pneumonia, suggesting that *T. whipplei* should be considered more as a pathogen when detected in BALF in immunocompromised patients with clinical symptoms. Mycobacterium tuberculosis complex and *T. whipplei* co-infection or detection were occasionally reported in other studies (Lagier et al., 2016; Zhu et al., 2021). However, our study found that Mycobacterium tuberculosis complex was the most common pathogenic bacteria detected together, and a considerable number of patients (34.3%) were diagnosed as reactivation pulmonary tuberculosis. The reason may be that the incidence of tuberculosis in China is still high. At the same time, our hospital was a designated hospital for tuberculosis treatment, which leaded to the increase of detection rate. On the other hand, both Mycobacterium tuberculosis complex and *T. whipplei* belong to *Actinobacteria*. Mycobacterium tuberculosis complex is facultative intracellular bacterium, and *T. whipplei* is obligate intracellular bacterium. The infection of both bacteria have been associated with macrophages. In the pathogenesis of classic WD, macrophages in duodenal mucosa can be induced by bacteria to polarize into M2/alternatively activated macrophages (Desnues B, Lepidi et al., 2005). The impaired antigen-presenting function of macrophages and dendritic cells further weakened the response of T cells (Moos et al., 2010). In addition, the increased activity of regulatory T cells in the gut and blood further increases the immunosuppressive environment (Schinnerling et al., 2011). However, due to the lack of research on the pathogenesis of *T. whipplei* in lung infection, we can only speculate that the co-infection or detection of Mycobacterium tuberculosis complex and *T. whipplei* may be related to the impairment of pulmonary macrophage function. The interaction and causality between *T. whipplei* and other pathogens in the lung need to be further studied.

We performed a general classification of chest CT findings in non-pulmonary tuberculosis patients, especially those considering *T. whipplei* as the dominant pathogen. Therefore, our results are representative and convincing. The overall chest CT findings were diverse, with pulmonary nodules as the main manifestation, which had also been mentioned in several previous case reports (Urbanski et al., 2012; Zhang and Xu, 2021). Nevertheless, according to the histopathological results, a considerable number of pulmonary nodules were identified as tumors, most of which were early-stage lung adenocarcinoma. In the long-term follow-up survey of WD patients with a small sample size (35 patients) by Schiepatti et al., 22% (7 patients) had preneoplastic/neoplastic disorders (Schiepatti et al., 2020). At present, the risk factors for the occurrence of pulmonary nodules are not clear. We speculate that there may be a relationship between lung lesions and *T. whipplei*. Whether the presence of *T. whipplei* in the lung microbiome may promote the occurrence of pulmonary nodules or even early-stage lung cancer directly or indirectly by changing the immune microenvironment still needs to be further confirmed by prospective studies with a larger sample size.

In this study, duodenal mucosal biopsy was performed in a small number of patients, and PAS staining was not performed in suspected patients, so WD was not clearly diagnosed. As WD is a multisystem disease, untreated WD is fatal (Marth and Raoult, 2003). Therefore, we believe that duodenal mucosal biopsy, PAS staining, and immunohistochemistry can be considered for patients suspected of *T. whipplei* infection to further improve the diagnosis. PCR in sterile body fluids, such as blood, cerebrospinal fluid, and joint fluid, can help diagnose WD, but most of them are invasive procedures, which still need to be evaluated according to the condition. This study also had limitations. It was a single-center retrospective descriptive study without longitudinal follow-up, and partial information was not comprehensive. No control group was established, so correlation and causality could not be determined. But we believe our study is informative.

## Conclusion

In summary, this study objectively analyzed the clinical characteristics of patients with positive *T. whipplei* detected by mNGS in BALF. Our study adds to the evidence that *T. whipplei* in BALF may be related to some lung diseases. *T. whipplei* should be considered as a potential risk factor for pneumonia when detected in immunocompromised patients. For non-immunocompromised patients, the detection of *T. whipplei* also needs attention because of the relationship between *T. whipplei* and pulmonary nodules. mNGS has improved the detection and attention of rare pathogens. Moreover, the infection, colonization, and prognosis of *T. whipplei* in the lung need further study.

## Data availability statement

The raw data supporting the conclusions of this article will be made available by the authors, without undue reservation.

## Ethics statement

The studies involving human participants were reviewed and approved by the Ethics Committee of the Fifth Affiliated Hospital of Sun Yat-Sen University (approve number [2022] K82-1). Written informed consent for participation was not required for this study in accordance with the national legislation and the institutional requirements.

## Author contributions

ML participated in data collection, analysis, and manuscript drafting. KW participated in drafting the work, acquisition, and analysis. LQ and YL participated in data sorting and input. CTu, MC, ZW, JW, YH, and CTa participated in sample collection. QC and XZ participated in the conception and design of the work. JL conceived of the study, and participated in its design, and helped to draft the manuscript. All authors contributed to the article and approved the submitted version.

## Funding

JL was grant from Guangdong Basic and Applied Basic Research Foundation (2020A1515011147). ML was supported by Open project of Key Laboratory of Tropical Disease Control (Sun Yat-sen University), Ministry of Education (2020kfkt04).

## Acknowledgement

We are grateful to BGI for their assistance in detection methodology.

## Conflict of interest

The authors declare that the research was conducted in the absence of any commercial or financial relationships that could be construed as a potential conflict of interest.

## Publisher’s note

All claims expressed in this article are solely those of the authors and do not necessarily represent those of their affiliated organizations, or those of the publisher, the editors and the reviewers. Any product that may be evaluated in this article, or claim that may be made by its manufacturer, is not guaranteed or endorsed by the publisher.
