# Supplementary figures and images for: Tropheryma whipplei detection by metagenomic next-generation sequencing in bronchoalveolar lavage fluid: A cross-sectional study
Source: Front Cell Infect Microbiol. 2022 Aug 17;12:961297. doi: 10.3389/fcimb.2022.961297 (PMC9428251; doi:10.3389/fcimb.2022.961297)

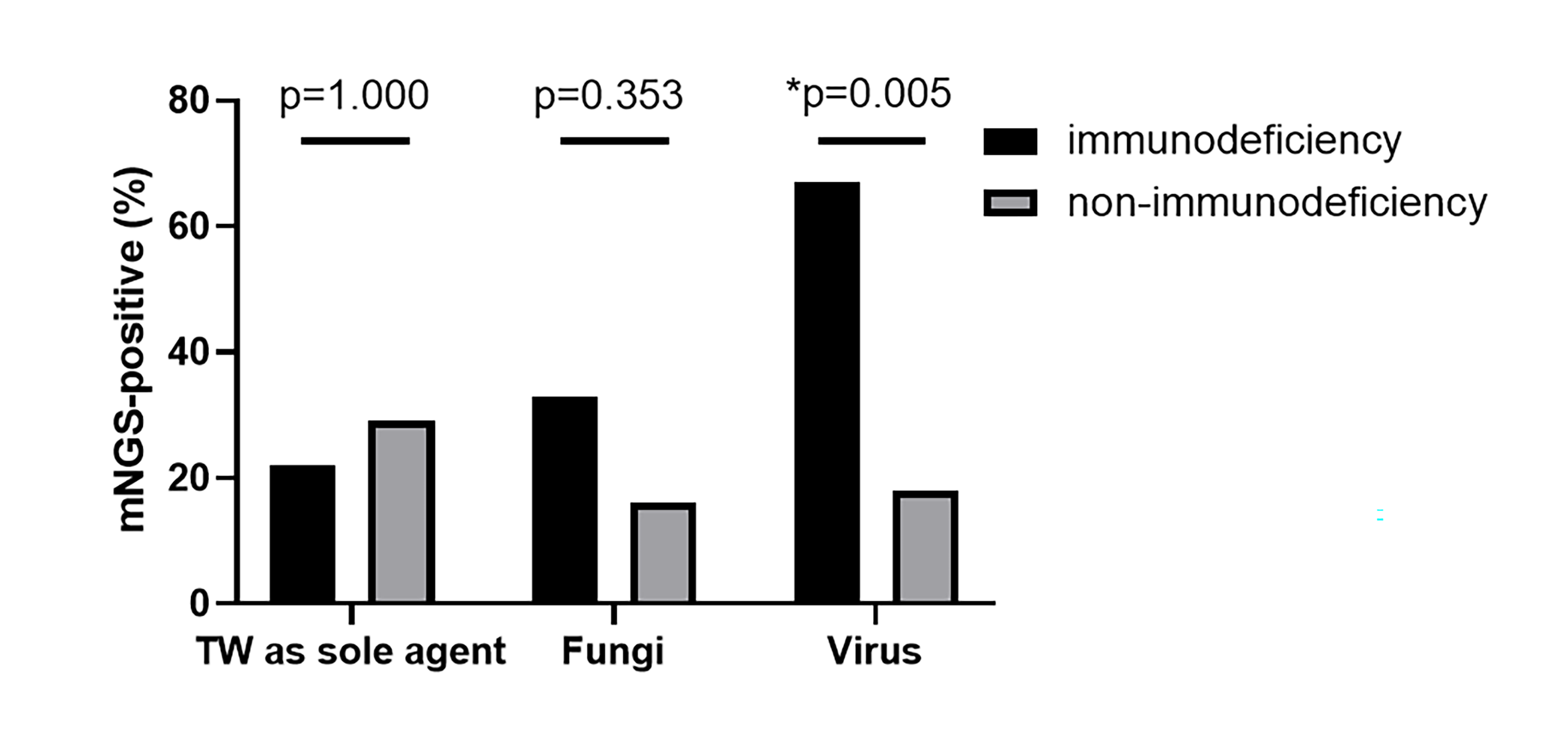


**Supplementary** **Figure S1.** Positive detection rate of mNGS associated with immune state.

Supplement: Supplementary file 1 [file DataSheet_1.docx]
